# Clinical Characteristics of Rapid Progression in Asia-Pacific Patients With ADPKD

**DOI:** 10.1016/j.ekir.2023.06.018

**Published:** 2023-06-26

**Authors:** Yun Kyu Oh, Hyunjin Ryu, Curie Ahn, Hayne C. Park, Yiyi Ma, Dechao Xu, Tevfik Ecder, Tze-Wah Kao, Jeng-Wen Huang, Gopala K. Rangan, RAPID-ADPKD Study Group, Yun Kyu Oh, Hyunjin Ryu, Curie Ahn, Hayne C. Park, Yiyi Ma, Dechao Xu, Changlin Mei, Tevfik Ecder, Angela Yee-Moon Wang, Tze-Wah Kao, Jeng-Wen Huang, Gopala K. Rangan

**Affiliations:** 1 Department of Internal Medicine, Seoul Metropolitan Government Seoul National University Boramae Medical Center, Seoul, Korea; 2 Department of Internal Medicine, Seoul National University Hospital, Seoul, Korea; 3 Department of Internal Medicine, Kangnam Sacred Heart Hospital, Seoul, Korea; 4 Department of Nephrology, Kidney Institute, Second Affiliated Hospital, Navy Medical University, Shanghai, China; 5 Department of Internal Medicine, Division of Nephrology, Faculty of Medicine, Istanbul Bilim University, Istanbul, Turkey; 6 Division of Nephrology, Department of Internal Medicine, Fu Jen Catholic University Hospital, Fu Jen Catholic University, New Taipei City, Taiwan; 7 Division of Nephrology, Department of Internal Medicine, National Taiwan University Hospital and National Taiwan University College of Medicine, Taipei, Taiwan; 8 Michael Stern Laboratory for Polycystic Kidney Disease, Westmead Institute for Medical Research, The University of Sydney and the Department of Renal Medicine, Westmead Hospital, Western Sydney Local Health District, Westmead, New South Wales, Australia

**Keywords:** Asia-Pacific region, autosomal dominant polycystic kidney disease, estimated glomerular filtration rate, height-adjusted total kidney volume, rapid progression

## Abstract

**Introduction:**

This study aimed to determine the utility of different methods to predict rapid progressors (RPs) and their clinical characteristics in Asia-Pacific patients with autosomal dominant polycystic kidney disease (ADPKD).

**Methods:**

This was a multinational retrospective observational cohort study of patients with ADPKD in the Asia-Pacific region. Five hospitals from Australia, China, South Korea, Taiwan, and Turkey participated in this study. RP was defined by European Renal Association–European Dialysis and Transplantation Association (ERA-EDTA) guidelines and compared to slow progressors (SPs).

**Results:**

Among 768 patients, 426 patients were RPs. Three hundred six patients met only 1 criterion and 120 patients satisfied multiple criteria for RP. Historical estimated glomerular filtration rate (eGFR) decline fulfilled the criteria for RP in 210 patients. Five patients met the criteria for a historical increase in height-adjusted total kidney volume (TKV). The 210 patients satisfied the criteria for based on kidney volume. During the follow-up period, cyst infections, cyst hemorrhage, and proteinuria occurred more frequently in RP; and 13.9% and 2.1% of RPs and SPs, respectively, progressed to end-stage kidney disease (ESKD). RP criteria based on historical eGFR decline had the strongest correlation with eGFR change over a 2-year follow-up

**Conclusion:**

Various assessment strategies should be used for identifying RPs among Asian-Pacific patients with ADPKD in real-world clinical practice during the follow-up period, cyst infections, cyst hemorrhage, and proteinuria occurred more frequently; and more patients progressed to ESKD in RPs compared with SPs.

## Graphical abstract

**Figure:**
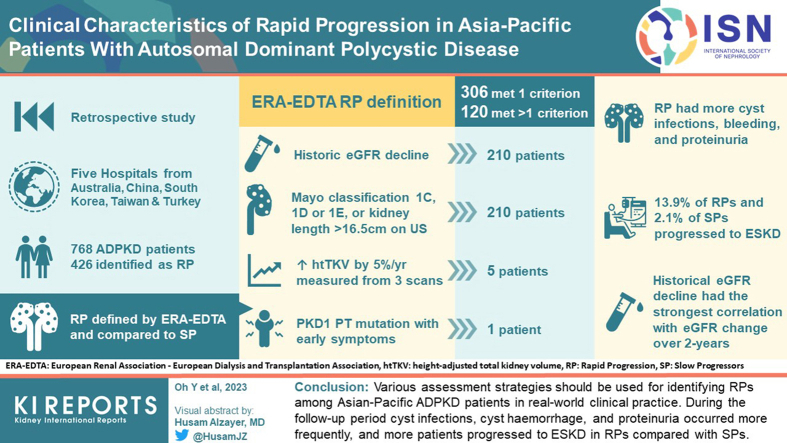

ADPKD is the most common hereditary kidney disease and is characterized by the development of numerous cysts and a gradual decline in kidney function, leading to kidney failure in approximately 50% of patients in their sixth decade.^1^^,^^2^ ADPKD is caused by mutations in the *PKD1* (85% of cases) and *PKD2* (15% of cases) genes.^3^^,^^4^ Recently, other genes, such as *GNANB* and *DNAJB11,* have also been identified as causative genes.^5^^,^^6^

ADPKD has a clinically heterogeneous phenotype, and the rate of renal function shows significant variability.^7^ Although this variability is primarily because of genetic diversity, it may also be influenced by other clinical characteristics and environmental factors.^8^ It is important to predict whether the patient’s kidney disease will progress rapidly to kidney failure and therefore to implement early treatment strategies to slow disease progression. After tolvaptan, a vasopressin V2 receptor antagonist, was approved as an effective drug for slowing the progression of ADPKD, it became more important to define patients who were RPs.^9^ Height-adjusted KTV (htTKV) indexed for age, represented by the Mayo imaging classification (MIC), was the most important factor in predicting a future decline in glomerular filtration rate in typical bilateral diffuse cystic kidney disease.^10^ Patients with *PKD1* truncating (*PKD1 PT*) mutations had a more aggressive course than those with *PKD1* nontruncating (*PKD1 NT*) or *PKD2* mutations.^11^ The predicting renal outcomes in ADPKD (PROPKD) score accurately predicted kidney outcome using 4 variables that were significantly associated with age at ESKD onset: male sex, hypertension before 35 years of age, first urologic event before 35 years of age, and *PKD* mutation type.^12^ The ERA-EDTA Working Groups of Inherited Kidney Disorders and European Renal Best Practice (WGIKD/ERBP) have provided algorithms defining rapid progression for initiation of treatment using historical eGFR decline and historical kidney growth. They also define likely rapid progression as MIC 1C, 1D, and 1E, ultrasound length >16.5 cm and/or truncating *PKD1* mutation + early symptoms (i.e., a PROPKD score >6). Finally, they define possible rapid progression by a family history of ADPKD that reaches ESKD at ≤58 years.^13^

However, most of these definitions and algorithms for defining RP were developed in Western countries, and it is not known whether they apply to the Asia-Pacific population. In addition, large-scale clinical studies such as the Tolvaptan Efficacy and Safety in Management of Polycystic kidney disease and outcomes 3:4 trial and the Replicating Evidence of Preserved Renal Function: an Investigation of Tolvaptan Safety and Efficacy in ADPKD trial were mostly conducted in the United States and Europe; Asia-Pacific patients were excluded from the studies.^9^^,^^14^ The genetic background, race, climate, culture and lifestyle, availability of genetic testing, and medical insurance system of this area are different from Western countries. Therefore, the retrospective epidemiologic study of Asian-Pacific patients with rapid disease progression of Autosomal Dominant Polycystic Kidney Disease (RAPID-ADPKD), a multinational retrospective observational cohort study of patients with ADPKD in the Asia-Pacific region, was developed.^15^ This study assessed the clinical utility of various assessment strategies to predict RPs based on the ERA-EDTA WGIKD/ERBP algorithm in patients enrolled in the RAPID-ADPKD study. The characteristics and outcomes of patients classified as either RPs or SPs were also investigated. This is the first multinational study to collect real-world clinical practice data from Asia-Pacific patients.

## Methods

### Study Design

The study protocol of the RAPID-ADPKD study has published elsewhere.^15^ Briefly, this was a multinational, retrospective observational medical chart review study from the Asia-Pacific region. Each site investigator completed an electronic case report form for eligible patients with ADPKD who were identified according to inclusion and exclusion criteria. Data were derived from case records from patients attending outpatient clinics.

### Study Site

The following 5 centers in the Asia-Pacific region participated in the study: (i) Westmead Hospital, Sydney, Australia; (ii) Changzheng Hospital, Shanghai, China; (iii) Seoul National University Hospital, Seoul, South Korea; (iv) National Taiwan University Hospital, Taipei, Taiwan; and (v) Bilim University, Istanbul, Turkey.

### Study Population

After retrospectively reviewing data for patients referred to each center, patients with ADPKD who were ≥18 years of age (≥19 years old for patients in Taiwan because of the different age criteria for adults in this country) and with an eGFR ≥30 ml/min per 1.73 m^2^ at the index date of the study were included for analysis in this study. The index date was defined as the first medical record reviewed for the purpose of this study. Patients were required to have at least 2 clinical visits with eGFR measurements and have been followed-up with at the investigational site for at least 24 months between January 2010 and the index date, and at least 2 years of clinical follow-up records from the index date. Enrolled patients were diagnosed with ADPKD according to the unified ultrasound criteria for patients with a family history of ADPKD.^16^ For patients without a family history of ADPKD, those with a clinical diagnosis of ADPKD based on typical radiological findings and/or clinical evaluation were also included.^15^

Patients with severe heart failure (symptoms of New York Heart Association class 3 and 4), severe liver disease (Child‒Pugh class B or C), chronic inflammatory disease, diabetic nephropathy, vascular disease and/or other comorbidities that can affect renal function were excluded based on the clinician’s judgment. Patients with active cancer who underwent chemotherapy, or with any medical or surgical conditions that could affect renal function or kidney volume were also excluded.^15^

This study was a retrospective, observational cohort study, and there were no interventions provided to the study subjects. All institutional review boards approved the consent waiver.

### Data Collection

Demographic information at the index date, such as age, sex, date of birth, race, height and body weight, and blood pressure, were collected. Comorbid conditions, including diabetes mellitus, hypertension, hyperuricemia, coronary artery disease, noncoronary heart disease, cerebrovascular disease, were investigated. Considering that the serum creatinine (Scr) measurement method varied across sites, the sites entered Scr values as well as the method utilized. eGFR was calculated based on the Chronic Kidney Disease Epidemiology Collaboration formula.^17^ If the Scr measurement method was not calibrated with isotope dilution mass spectrometry, the recorded Scr values were reduced by 5% before entry into the Chronic Kidney Disease Epidemiology Collaboration formula.^18^ Family history of ESKD, medications and other laboratory results were also collected. At the index date and during the follow-up visits, Scr; urine protein-to-creatinine ratio; htTKV from radiological findings on magnetic resonance imaging, computed tomography or ultrasound sonography; and ADPKD-related kidney complications were investigated. MIC was defined on the basis of annual htTKV growth rate estimated from patient’s age and a theoretical starting htTKV (1A <1.5%, 1B 1.5 to <3.0%, 1C 3.0 to <4.5%, 1D 4.5–6·.0%, and 1E >6.0%:).^10^ PROPKD scores were calculated based on the following: a score of 1 for men, a score of 2 for hypertension before 35 years of age, a score of 2 for having a first urological event such as macroscopic hematuria, flank pain or cyst infection before 35 years of age, a score of 2 for *PKD1 NT* mutation, and a score of 4 for *PKD1 PT* mutation based on genetic testing results.^12^^,^^15^

### TKV Calculation

To collect the maximum amount of htTKV data, we gathered data from renal magnetic resonance imaging, computed tomography, or ultrasound images obtained during the follow-up period. If the htTKV had already been measured using imaging, the value and the measurement methods for TKV (ellipsoid, stereological measurement, or other) were collected. For the analysis, htTKVs calculated by ellipsoid methods (length × width × depth × π/6) were used mainly to define rapid progression.^15^

### Definition and Outcome Variables

Rapid progression was defined when any of the following criteria were met based on European Renal Association–European Dialysis and Transplantation Association WGIKD/ERBP recommendations: (i) Historical eGFR decline: an annual eGFR decline ≥5 ml/min per 1.73 m^2^ within 1 year and/or ≥2.5 ml/min per 1.73 m^2^ per year over a period of 5 years; (ii) an increase in htTKV ≥5% per year measured from ≥3 radiological images; (iii) Mayo classification 1C, 1D or 1E, or kidney length of >16.5 cm on ultrasonography; and (iv) *PKD1 PT* mutation with early symptoms (PROPKD score >6). The remainder of the patients were classified as SPs. After dividing the patients into RP and SP groups, the clinical characteristics and renal disease-related characteristics of each group were described. ADPKD-related kidney complications, eGFR change, and ESKD during follow-up were collected.

### Statistical Methods

The baseline characteristics and laboratory data are presented as the means ± SD for continuous variables, and as frequencies and percentages for categorical variables. For the primary analysis, all clinical variables were analyzed according to 2 categories: rapid progression and slow progression. The difference in proportions among subgroups was tested with the chi-squared test; the mean among subgroups was analyzed with a T-test or analysis of variance. To examine relationship between changes in eGFR with baseline and disease characteristics, generalized linear mixed models were used to compare changes in eGFR over the 2-year follow-up. The Cox proportional hazards model was used to analyze the time-to-event (i.e., ESKD). A *P-*value < 0.05 was interpreted as statistically significant. All statistical analyses were performed using SAS software, version 9.4 (SAS Institute Inc., Cary, NC, USA).

## Results

### Clinical Characteristics of the Study Population

**Figure 1:**
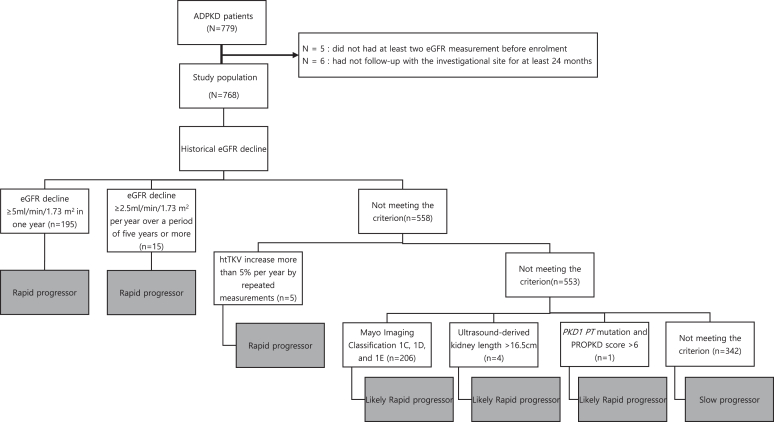
Patients eligible for rapid progressor status according to stepwise application of strategies based on ERA-EDTA WGIKD/ERBP recommendations. eGFR, estimated glomerular filtration rate; htTKV, height-adjusted total kidney volume; *PKD1 PT*, *PKD1* protein truncating

A total of 779 subjects were screened for study eligibility. Eleven patients were excluded from the study (*n* = 5 did not have at least 2 eGFR measurements before enrolment; *n* = 6 patients were lost to follow-up for at least 24 months. Therefore, 768 patients were assessed (Figure 1). The origin of patients is shown in Table 1 (*n* = 71 Turkey; *n* = 107 Taiwan; *n* = 300 Korea; *n* = 90 China; *n* = 200 Australia). The average follow-up duration after the index date was 3.28 ± 1.30 years, and the average number of visits was 7.0 ± 4.4 during follow-up. Detailed demographic, clinical and kidney disease-related characteristics of patients across sites are stated in Supplementary Tables S1 and S2.

**Table 1:** Patients eligible for rapid progressor status according to strategies based on ERA-EDTA WGIKD/ERBP recommendation

Criterion for RP ADPKD	All patients (*N* = 768)	Turkey (*N* = 71)	Taiwan (*N* = 107)	Korea (*N* = 300)	China (*N* = 90)	Australia (*N* = 200)
Total RPs, *n* (%)	426 (55.5)	34 (47.9)	25 (23.4)	200 (66.7)	73 (81.1)	94 (47.0)
eGFR decline in 1 year^a^, *n* (%)	195 (25.4)	21 (29.6)	23 (21.5)	37 (12.3)	61 (67.9)	53 (26.5)
eGFR decline over 5 years^b^, *n* (%)	38 (5.0)	7 (9.9)	0 (0.0)	4 (1.3)	20 (22.2)	7 (3.5)
Historical htTKV growth^c^, *n* (%)	7 (0.9)	0 (0.0)	0 (0.0)	7 (2.3)	0 (0.0)	0 (0.0)
Mayo imaging classification 1C-1E, *n* (%)	274 (35.7)	12 (16.9)	0 (0.0)	188 (62.8)	24 (26.7)	50 (25.0)
Kidney length (>16 cm), *n* (%)	6 (0.8)	1 (1.4)	2 (1.9)	0 (0.0)	0 (0.0)	3 (1.5)
*PKD1 PT* mutation and PROPKD score (>6), *n* (%)	39 (5.1%)	0 (0.0)	0 (0.0)	39 (13.0)	0 (0.0)	0 (0.0)

ADPKD, autosomal dominant polycystic kidney disease; htTKV, height-adjusted total kidney volume; *PKD1 PT*, *PKD1* protein truncating; RP, rapid progressor.

%, proportion of patients to total number of eligible patients.

a ≥5 ml/min per 1.73 m^2^.

b ≥2.5 ml/min per 1.73 m^2^/yr.

c htTKV ≥5%/yr.

The patients were predominantly middle-aged (47.1 ± 12.4 years) and 46.0% were female. The mean systolic and diastolic blood pressure at index was 131.1 ± 15.3 and 83.2 ± 10.7 mmHg, respectively**.** Of the patients, 37.9% had a family history of ADPKD reaching ESKD, 40.1% did not and 22.0% were unaware. Preexisting comorbidities of eligible patients include hypertension (80.1%), hyperuricemia (20.1%), cerebrovascular disease (4.3%), and noncoronary heart disease (3.4%) among others. A minority of patients (14.6%) had no preexisting comorbidities (Table 2).

**Table 2:** Clinical characteristics of patients at the index date

Variables	All patients *N* = 768	RP *N* = 426	SP *N* = 342	*P* value
Age (yrs)	47.1 ± 12.4	45.5 ± 11.3	49.1 ± 13.3	<0.01
Male (%)	398 (51.8)	234 (54.9)	164 (48.0)	0.05
BMI (kg/m^2^)	24.5 ± 4.3	24.9 ± 4.4	24.0 ± 4.1	0.01
Systolic BP (mm Hg)	131.1 ± 15.3	131.5 ± 15.5	130.6 ± 15.1	0.46
Diastolic BP (mm Hg)	83.2 ± 10.7	84.0 ± 10.1	82.1 ±1.3	0.02
DM (%)	5.1	4.0	6.7	0.09
Family history of ADPKD reaching ESKD (%)				<0.01
Yes	291 (37.9)	189 (44.4)	102 (29.8)	
No	259 (40.1)	165 (38.7)	143 (41.8)	
Unknown	146 (22.0)	72 (16.9)	97(28.4)	
Pre-existing comorbidities (%)				
None	112 (14.6)	50(11.7)	62(18.1)	0.01
Hypertension	615 (80.1)	366 (85.9)	249 (72.8)	<0.01
Hyperuricemia	154 (20.1)	107 (25.1)	47 (13.7)	<0.01
Coronary artery disease	19 (2.5)	7 (1.7)	12 (3.5)	0.10
Noncoronary heart disease	26 (3.4)	8 (1.9)	18 (5.3)	0.01
Cerebrovascular disease	33 (4.3)	16 (3.8)	17 (5.0)	0.41
No concomitant medication	67 (8.7)	23 (5.4)	44 (12.9)	<0.01
Antihypertensive medication	604 (78.7)	365 (85.7)	239 (69.9)	<0.01
ARBs	447 (58.2)	270 (63.4)	177 (51.8)	<0.01
ACE inhibitors	117 (15.2)	64 (15.0)	53 (15.5)	0.86
DPH CCBs	281 (6.6)	180 (42.3)	101 (29.5)	<0.01
Non-DPH CCBs	18 (2.3)	11 (2.6)	7 (2.1)	0.63
Beta blockers	184 (24.0)	124 (29.1)	60 (17.5)	<0.01
Diuretics	52 (6.8)	24 (5.6)	28 (8.2)	0.16
Uric acid-lowering agents	161 (21.0)	115 (27.0)	46 (13.5)	<0.01
Lipid-lowering agents	215 (28.0)	120 (28.2)	95 (27.8)	0.90

ACE, angiotensin converting enzyme; ADPKD, autosomal dominant polycystic kidney disease; ARBs, aAngiotensin II receptor blockers; BMI, body mass index; BP, blood pressure; DM, diabetes mellitus; DPH CCBs, dDihydropyridine calcium channel blockers; ESKD, end-stage kidney disease; RP, rapid progressor; SP, slow progressor.

Two hundred twenty-nine (29.8%) patients were with chronic kidney disease (CKD) stage 1; 259 (33.7%) were with CKD stage 2; 134 (17.5%) were with CKD stage 3a; and 146 (19.0%) were with CKD stage 3b (Table 3). The mean serum creatinine level was 1.12 ± 0.40 mg/dl and mean eGFR was 68.3 ± 28.4 ml/min per 1.73 m^2^. The mean urinary protein creatinine ratio was 160.6 ± 310.9 mg/mg in the overall population. MIC was reported for 394 eligible patients except those in Taiwan; overall, most patients had MIC 1C (37.8%), followed by 1B (25.8%) and 1D (20.8%). Kidney length by ultrasound was not reported in Korea and China. The genotype was determined for 256 patients, and the PROPKD score was calculated completely for 181 patients among which most patients were from Korea (Table 3 and Supplementary Table S2).

**Table 3:** Kidney disease-related characteristics of patients at the index date

Variables	All patients *N* = 769	RP *N* = 426	SP *N* = 342	*P* value
Serum creatinine (mg/dl)	1.12 ± 0.40	1.18 ± 0.40	1.04 ± 0.38	<0.01
eGFR (ml/min per 1.73 m^2^)	68.3 ± 28.4	64.6 ± 27.5	72.9 ± 29.0	<0.01
Blood urea nitrogen (mg/dl)	18.9±14.3	19.7±17.4	17.6±7.6	0.08
Uric acid (mg/dl)	5.7±1.6	5.9±1.5	5.4±1.6	<0.01
Urine PCR (mg/mg)	160.6 ± 310.9	188.9 ± 341.6	114.3± 305.4	0.01
CKD stage (%)				<0.01
1	229 (29.8)	107 (25.1)	122 (35.7)	
2	259 (33.7)	141 (33.1)	118 (34.5)	
3a	134 (17.5)	77 (18.1)	57 (16.7)	
3b	146 (19.0)	101 (23.7)	45 (13.2)	
Mayo imaging classification	*N* = 394	*N* = 285	*N* = 109	<0.01
Class 1A	26(6.6)	3(1.1)	23(21.1)	
Class 1B	101(25.6)	15(5.3)	86(78.9)	
Class 1C	149(37.8)	149(52.3)	0(0.0)	
Class 1D	8(20.8)	82(28.8)	0(0.0)	
Class 1E	36(9.1)	36(12.6)	0(0.0)	
HtTKV(ml/m)	1002.8 ± 664.8	1211.6 ± 667.4	473.3 ± 207.6	<0.01
Kidney length by ultrasound (cm)	13.5 ± 3.1 (*N* = 50)	15.3 ± 3.0 (*N* = 17)	12.6 ± 2.7 (*N* = 33)	<0.01
Genetic test results	*N* = 256	*N* = 181	*N* = 75	
*PKD1 PT* mutation	118(46.1)	95(52.5)	23(30.7)	<0.01
*PKD1 NT* mutation	66(25.8)	45(24.9)	21(28.0)	0.03
PKD2 mutation	48(18.8)	29(16.0)	19(25.3)	0.48
Mutation not found	24(9.4)	12(6.6)	12(16.0)	0.58
Unknown	612(66.7)	245(63.6)	267(78.1)	<0.01
PROPKD score	*N* = 181	*N* = 135	*N* = 46	<0.01
0–3	64 (35.4)	38(28.1)	26(56.5)	
4–6	70(38.7)	50(37.0)	20(43.5)	
7–9	47(26.0)	47(34.8)	0(0)	
Missing	587(76.4)	292(68.5)	292(86.3)	<0.01

CKD, chronic kidney disease; eGFR, estimated glomerular filtration rate; htTKV, height-adjusted total kidney volume; PCR, protein-to-creatinine ratio; *PKD1 NT*, *PKD1* nontruncating; *PKD1 PT*, *PKD1* protein truncating; RP, rapid progressor; SP, slow progressor.

### Classification of Rapid Progression by ERA-EDTA WGIKD/ERBP Recommendation

#### ERA-EDTA WGIKD/ERBP Method

A total of 426 patients were RPs. Historical eGFR: an annual eGFR decline ≥5 ml/min per 1.73 m^2^ within 1 year and ≥2.5 ml/min per 1.73 m^2^ per year over a period of 5 years fulfilled the criteria for rapid progression in 195 patients and 38 patients, respectively. Seven patients met the criteria for a historical increase in htTKV of more than 5% per year by repeated measurements. The 274 patients with MIC 1C, 1D, or 1E; and the 6 patients with kidney length >16.5 cm were considered likely to be RP. Thirty-nine patients were likely to be RP based on a *PKD1 PT* mutation and PROPKD score >6 (Table 1). Three hundred six patients met only 1 criterion and 120 patients satisfied multiple criteria for RP (Supplementary Table S3).

The method for RP assessment varied by country. In Turkey, RPs were diagnosed in order of eGFR decline in 1 year, MIC, and eGFR decline over 5 years criteria; in Taiwan in order of eGFR decline in 1 year and kidney length criteria; in Korea in order of MIC, *PKD1 PT* mutation and PROPKD score, and eGFR decline in 1 year, in China in order of eGFR decline in 1 year, MIC, and eGFR decline over 5 years criteria; and in Australia eGFR decline in 1 year, MIC, and eGFR decline over 5 years criteria (Table 1).

#### Stepwise Method on the ERA-EDTA WGIKD/ERBP Recommendation

In total, 210 patients met the criteria for RP based on historical eGFR decline. Of the 558 patients remaining, 5 fulfilled the RP criteria according to historical htTKV growth. Of the remaining 553 patients, 206 patients were considered to have likely RP based on MIC 1C, 1D, or 1E. In addition, 4 patients were considered to have likely RP based on ultrasound-derived kidney length >16.5 cm. The 343 patients remaining were assessed for likely RP using the PROPKD score, and 1 patient was categorized as likely RP. A total of 342 patients did not meet any of the above criteria and were categorized as having slow progression (Figure 1). The proportion of RPs varied from country to country. In China, Korea, Turkey, Australia, and Taiwan, 81.1, 66.7, 47.9, 47.0, and 23.5% of eligible patients, respectively, were RPs (Table 1).

### Characteristics of RPs and SPs

#### Demographics

RPs were younger (45.5 ± 11.3 vs. 49.1 ± 13.3 years, *P* < 0.01), and had higher body mass index (24.9 ± 4.4 vs. 24.0 ± 4.1 kg/m^2^, *P* = 0.01) and higher diastolic blood pressures (84.0 ± 10.1 vs. 82.1 ±1.3 mmHg, *P* = 0.02). RPs had a greater incidence of CKD stage 3b (23.7% vs. 13.2%, *P* < 0.01) and a greater incidence family history of ADPKD reaching ESKD (44.4% vs. 29.8%, *P* < 0.01). They also had a greater incidence preexisting hypertension (85.9% vs. 72.8%, *P* < 0.01) and hyperuricemia (25.1% vs. 13.7%, *P* < 0.01) (Table 2). Compared with the RPs, there was a statistically significant greater proportion of patients who were not prescribed concomitant medications, among the SPs (5.8% vs. 12.5%, *P* < 0.001). A greater proportion of patients were prescribed antihypertensive agents among the RPs than among SPs (85.2% vs. 70.2%, *P* < 0.0001), of which the antihypertensive agents included dihydropyridine calcium channel blockers (41.9% vs. 29.8%, *P* < 0.001), angiotensin II receptor blockers (63.2% vs. 51.8%, *P* = 0.001) and beta blockers (28.9% vs. 17.6%, *P* < 0.0001) (Table 2)

#### Genetic Analysis

Genetic analysis was performed in 256 patients; 181 had rapid progression, and 75 had slow progression. RPs had more *PKD1 PT* mutations than SPs (52.5% vs. 30.7%, *P* < 0.01); however, 47.5% of RPs had *PKD1 NT, PKD2* or unidentified mutations (Table 3). The PROPKD score was calculated in 181 patients; 135 were RPs, and 46 patients were SPs. Among 135 RPs, 47 (34.8%) patients had PROPKD scores >6, and 88 (65.2%) patients had scores of 0 to 6 (Table 3).

#### PROPKD Score

Compared with the SPs, there were statistically significant differences in proportions of patients who had PROPKD scores of 5, 6, and 7 in the RPs. Compared with the SPs, there were higher proportions of patients who had hypertension before 35 years of age, first urologic event of macroscopic hematuria, cyst infection before 35 years of age, *PKD1 PT* mutation, and *PKD1 NT* mutation in the RPs (Table 3).

#### Kidney Disease Outcomes

RPs had lower eGFRs (64.6 ± 27.5 vs. 72.9 ± 29.0 ml/min per 1.73 m^2^, *P* < 0.01), higher uric acid levels (5.9 ± 1.5 vs. 5.4 ± 1.6 mg/dl, *P* < 0.01), and higher urinary protein-to-creatinine ratios (188.9 ± 341.6 vs. 114.3 ± 305.4 mg/mg, *P* = 0.01) (Table 3). A total of 285 RPs and 109 SPs had htTKV data, and their htTKVs were 1211.6 ± 667.4 and 473.3 ± 207.6 ml/m, respectively (*P* < 0.01). Among 285 RPs, 3 and 15 patients were classified as MIC 1A and 1B, respectively (Table 3). During the follow-up period, 13.9% and 2.1% of RPs and SPs progressed to ESKD, respectively (*P* < 0.01) (Table 4). Multivariate analysis with generalized linear mixed models showed that historical eGFR decline, MIC 1D–1E, and age group ≥51 years were associated with eGFR change over 2-year follow-up and a historical eGFR decline was the most important factor (Table 5). When time-to event analyses was performed using Cox proportional hazards model, RP, younger age, and higher systolic blood pressure had an association with risk of ESKD (Supplementary Table S4).

**Table 4:** Kidney complications and outcomes of rapid progression and slow progression during the follow-up period

Variables	RP (*N* = 426)	SP (*N* = 342)	*P* value
Renal event (%)			
Cyst infection	33 (7.7)	7 (2.0)	<0.01
Cyst hemorrhage	26 (6.1)	5 (1.4)	<0.01
Proteinuria	102(23.9)	53(15.5)	<0.01
Kidney stones	49(11.5)	3710.8)	0.93
Gross hematuria	44(10.3)	23(6.7)	0.12
Chronic pain	19(4.5)	14(4.1)	0.94
Upper UTI	13(3.1)	7(2.0)	0.45
Outcome events (%)			
Progressed to ESKD	59 (13.9)	7 (2.1)	<0.01

ESKD, end-stage kidney disease; RP, rapid progressor; SP, slow progressor.

**Table 5:** Multivariate analysis of change in eGFR over 2-year follow-up with generalized linear mixed models

Variables	Coefficient	95% CI		*P* value
Historical eGFR decline^a^	−20.2	−25.1	−15.3	0.001
MIC (Ref: 1A–1B)				
1C	−3.1	−6.8	0.6	0.078
1D–1E	−4.9	−9.3	−0.6	0.034
Age groups (Ref: ≤ 30 yrs)				
31–40	−3.5	−8.6	1.7	0.179
41–50	−4.8	−10.1	0.6	0.075
≥ 51	−6.9	−12.8	−1.1	0.025

CI, confidence interval; eGFR, estimated glomerular filtration rate; MIC, mayo imaging classification.

Adjusted for Historical eGFR decline, MIC, age group, chronic kidney disease stage, gender, family history of ADPKD reaching end-stage kidney disease, systolic blood pressure diastolic blood pressure, diabetes mellitus, *PKD* mutation

a eGFR decline <5 ml/min/1.73 m^2^ in one year or <2.5 ml/min/1.73 m^2^ in per over 5 years.

#### ADPKD Complications

During the follow-up period, cyst infections and cyst hemorrhage were observed at rates of 7.7% and 6.1% in RPs compared with 2.0% and 1.4% in SPs (*P* < 0.01 and *P* < 0.01, respectively). Proteinuria occurred at a rate of 23.9% among RPs compared with 15.5% among SPs (*P* < 0.01). The incidences of urinary stones, gross hematuria, chronic pain, and upper urinary tract infection, however, were not different between RPs and SPs (Table 4).

## Discussion

Identifying RPs in the treatment of patients with ADPKD is important not only for selecting patients who may benefit from tolvaptan but also for identifying high-risk patients who should be more carefully monitored.

Several models for predicting the progression of kidney in patients with ADPKD are available based on previous cohort studies. The Toronto Genetic Epidemiology Study of PKD emphasized the importance of genotype^11^ whereas the Mayo Clinic Translational Polycystic Kidney Disease Center is centered on volume assessment of the kidney,^10^ and the GENCYST study developed the PROPKD score.^12^ However, there is limited evidence for the stepwise evaluation of RP and renal prognosis of ADPKD. Furlano *et al.*^19^ analyzed 73 Spanish patients with ADPKD using the ERA-EDTA WGIKD/ERBP recommendations, and found that 19 patients met the RP criteria based on historical eGFR decline, 9 were considered to be RPs based on ultrasound kidney length >16.5 cm, and 20 were categorized as high-risk for RP based on MIC (1C, 1D, and 1E).^19^ Comparable to the current study, Furlano *et al.*^19^ reported that the proportion of RPs by historical eGFR decline was similar to that of MIC.

In the present study, the rate of eGFR decline, rate of htTKV growth, MIC, kidney length by ultrasound, genetic testing, family history, and PROPKD score were used to assess the risk of RP. Each country tends to apply various combinations of these criteria for selecting RP for tolvaptan treatment according to their local regulatory and clinical practice guidelines. In the United Sates, RPs are identified primarily by MIC, whereas only historical eGFR decline is used in Australia. In Japan, RP is defined only by TKV. In addition, the age and eGFR at treatment initiation are also set differently in each country.^20^ Other consensus or regulatory guidelines (Canadian Expert Consensus, PBS Australia, ERA-EDTA, NICE and Edinburgh Renal Unit guidelines) have used historical eGFR decline to identify the RPs and consideration of tolvaptan commencement.^20^ Although measurement of eGFR is widely accessible, it may be of limited value for predicting disease progression during the early stages of ADPKD when the eGFR decline is nonlinear, with a period of relative stability in most participants, followed by an accelerating decline.^21^ In the current study, 210 of 426 RPs met the criteria of historical eGFR decline, whereas the remainder (*n* = 216) were diagnosed by kidney volume criteria or PROPKD score, suggesting that a combined approach is required in some subpopulations of ADPKD.

In general, TKV typically increases continuously and quantifiably from the early stages of the disease and is associated with a decline in kidney function.^22^ Although TKV measurement is an objective indicator that can predict disease progression from the early disease stages, the proportion of patients who regularly undergo TKV measurement is low in real-world clinical practice. In our study, only 7 of 426 RPs were diagnosed by historical htTKV growth criteria.

Clinical practice in the United States and the National Health Insurance Service of Korea use MIC (subclasses 1C, 1D, and 1E) alone (without the need for additional markers) to define RPs and high-risk patients who should be treated with tolvaptan. However, in this study, MICs were obtained from only 394 of 768 patients. Among them, 274 patients were RPs. These results suggest that although MIC is an important prognostic factor, it is not always used in clinical practice possibly because of several disadvantages (high cost of magnetic resonance imaging, radiation exposure of computed tomography, time required for MIC calculation). In addition, 18 RPs for whom MICs were determined were classified as either subclass 1A or 1B. Although these 18 patients had relatively smaller kidneys than the other RPs, their historical eGFR decline or historical TKV growth was rapid. Further studies on the clinical and genetic characteristics of these patients are needed. Bhutani *et al.* suggested that an ultrasound kidney length >16.5 cm could stratify the risk of progression to renal insufficiency.^23^ However, ultrasound kidney length data were also only available in very few patients.

Although genetic testing provides prognostic information for ADPKD, it is not used in routine clinical practice because it is expensive, labor-intensive, and in most clinical situations, the diagnosis has been made with renal ultrasound using the Pei-Ravine criteria.^24^ In addition, to limited availability of genetic data, the PROPKD score may have limited value in retrospective studies, because obtaining a history of hypertension or urological symptoms was not systematic. Therefore, in this study, only 6.1% of patients had a PROPKD score >6, and 1 patient was additionally diagnosed with RP through the stepwise application of the algorithm.

During the follow-up period, cyst infection, cyst hemorrhage, proteinuria, and progression in ESKD were more frequent in RPs than SPs. Sallée *et al.*^25^ reported that 29 of 389 patients with ADPKD had 311 episodes of kidney cyst infection between January 1998 and August 2008. Ronsin *et al.*^26^ reported that 21 of 296 patients with ADPKD with renal allografts experienced 22 episodes of cyst infection over a median follow-up of 4 (2–7) years. The cumulative incidence rate was 3% at 1 year, 6% at 5 years, and 12% at 10 years posttransplantation. However, whether cyst infection is common among RPs has not been elucidated. It is possible that the incidence of cyst hemorrhage is higher in RPs because cyst hemorrhage may be related to rapid cystic growth or cyst infection. However, the risk factors for cyst infection and cyst hemorrhage have not been studied; therefore, more research is needed to determine whether cyst infection or cyst hemorrhage occurs more frequently in RPs and affects renal function deterioration. Although there is no consensus regarding whether proteinuria is a marker of disease progression, it has been shown to correlate with TKV and blood pressure in ADPKD.^27^ In this study, proteinuria was more frequently found during the follow-up period in RPs, but it was not revealed whether it was related to disease progression after adjustment for hypertension or htTKV.

Because ADPKD patients have relatively high eGFR values for their age group, the ERA-EDTA WGIKD/ERBP panel recommendation for tolvaptan treatment divided patients with eGFR indexed for age. The panel excluded patients aged 40–50 years with CKD stages 1 and 2 or patients aged 30 to 40 years with CKD stage 1 as candidates for tolvaptan treatment. The panel also excluded patients aged >50 years with a CKD stages 1 to 3a because it was considered likely that these patients have a high probability of slowly progressive disease.^13^ However, the Replicating Evidence of Preserved Renal Function: an Investigation of Tolvaptan Safety and Efficacy in ADPKD trial allowed for an extension of eligibility criteria to older patients and later-stage ADPKD.^14^ The importance of functional status rather than biological age is gaining attention in patient treatment. In addition, tolvaptan has not yet been approved in some countries in the Asia-Pacific area. Therefore, in this study, RP was selected as the criterion for omitting the eGFR indexed by age.

The main strength of this study is that it is based on real-world data from the clinical practice site of patients with ADPKD in the Asia-Pacific area and various assessment strategies were applied for identifying RPs. However, this study has several limitations. First, because this is a retrospective study; data was incomplete and some data were missing. In particular, there were no data on family history of reaching ESKD before the age of 58 years. Second, even within the Asia-Pacific area, each country has different family and social attitudes toward genetic disorders as well as policy and insurance coverage for testing or treatment. These differences could not be adjusted, and there may be differences in the accuracy and quality of the data collected. Finally, ERA-EDTA WGIKD/ERBP updated the recommendation for tolvaptan treatment in 2021.^28^ Because the RAPID-ADPKD study was designed in 2017, the results of the present study do not reflect the revised ERA-EDTA WGIKD/ERBP recommendations.

In conclusion, various assessment strategies should be used for identifying RPs among Asian-Pacific patients with ADPKD in real-world clinical practice. In the RPs defined in this way, the rate of eGFR decline was rapid, more patients progressed to ESKD, and there were more cases of cyst infections, cyst hemorrhage, and proteinuria compared with SPs.

## Disclosure

OYK has been site investigator in clinical trials sponsored by Otsuka Korea and reports speaker fees, reimbursement of travel and accommodation costs to attend RAPID-ADPKD Investigator meetings sponsored by Korea Otsuka International Asia Arab. CA has been site investigator in clinical trials sponsored by Otsuka Korea and reports speaker fees, reimbursement of travel and accommodation costs to attend RAPID-ADPKD Investigator meetings sponsored by Korea Otsuka International Asia Arab. GR has been site investigator in clinical trials sponsored by Otsuka Australia and reports speaker fees, reimbursement of travel and accommodation costs to attend RAPID-ADPKD Investigator meetings were sponsored by Korea Otsuka International Asia Arab. HR, HCP, YM, DX, TE, TWK, JWH have reported no conflicting of interests.

## Appendix

List of the RAPID-ADPKD Study Group

Yun Kyu Oh, Hyunjin Ryu, Curie Ahn, Hayne C. Park, Yiyi Ma, Dechao Xu, Changlin Mei, Tevfik Ecder, Angela Yee-Moon Wang, Tze-Wah Kao, Jeng-Wen Huang, and Gopala K. Rangan.

## Supplementary Materials

Supplementary File (PDF)

**Table S1.** Demographic and clinical characteristics of patients across sites.

**Table S2.** Kidney disease-related characteristics of patients across sites.

**Table S3.** Criterion for patients deemed to be in rapid progressor.

**Table S4.** Cox proportional hazards model for outcome event (end-stage kidney disease).
