# Supplementary material for: Clinical Characteristics of Rapid Progression in Asia-Pacific Patients With ADPKD
Source: Kidney Int Rep. 2023 Jun 26;8(9):1801–10. doi: 10.1016/j.ekir.2023.06.018 (PMC10496076; doi:10.1016/j.ekir.2023.06.018)
Supplement: Supplementary File (PDF) [file mmc1.docx]

**TABLES**

**Table S1.** Demographic and clinical characteristics of patients across sites

| Variables | **Turkey**  **(N=71)** | **Taiwan**  **(N=107)** | **Korea**  **(N=300)** | **China**  **(N=90)** | **Australia**  **(N=200)** |
| --- | --- | --- | --- | --- | --- |
| Age – years | 42.7 ± 10.6 | 50.2 ± 13.8 | 49.3 ± 11.5 | 44.2 ± 9.5 | 45.1± 13.4 |
| Age groups (%) |  |  |  |  |  |
| ≤30 | 5(7.0) | 6(5.6) | 18(6.0) | 3(3.3) | 29 (14.5) |
| 31 – 40 | 28 (39.4) | 20 (18.7) | 49 (16.3) | 33 (36.7) | 48 (24.0) |
| 41 – 50 | 22 (31.0) | 32 (29.9) | 90 (30.0) | 30 (33.3) | 56 (28.0) |
| ≥51 | 16 (22.5) | 29 (45.8) | 143 (47.7) | 24 (26.7) | 67 (33.5) |
| Sex (%) |  |  |  |  |  |
| Female | 29 (40.9) | 59 (55.1) | 153(51.0) | 37 (41.1) | 92 (46.0) |
| Male | 42 (59.2) | 48 (44.9) | 147 (49.0) | 53 (58.9) | 108 (54.0) |
| Ethnicity – n (%) |  |  |  |  |  |
| Asian | 0(0.0) | 107(100.0) | 300(100.0) | 90(100.0) | 26 (13.0) |
| Black | 0(0.0) | 0(0.0) | 0(0.0) | 0(0.0) | 0(0.0) |
| White | 70 (98.6) | 0(0.0) | 0(0.0) | 0(0.0) | 85 (42.5) |
| Others | 1(1.4) | 0(0.0) | 0(0.0) | 0(0.0) | 14(7.0) |
| Missing | 0(0.0) | 0(0.0) | 0(0.0) | 0(0.0) | 75 (37.5) |
| BMI – kg/m^2^ | 25.5 ± 3.9 | 23.6 ± 4.2 | 23.5 ± 3.0 | 25.2 ± 8.5 | 27.3 ± 4.9 |
| Systolic BP (mmHg) | 139.8 ± 15.8 | 133.0 ± 16.7 | 128.5 ± 12.1 | 130.7 ± 17.5 | 131.2 ± 16.8 |
| Diastolic BP (mmHg) | 88.3 ± 8.6 | 80.9 ± 11.2 | 83.5 ± 9.5 | 84.8 ± 10.8 | 80.9 ± 12.0 |
| DM – n (%) | 1 (1.4) | 18 (16.8) | 13 (.3) | 1 (1.1) | 7 (3.5) |
| Family history of ADPKD reaching ESRD – n (%) | | | | | |
| Yes | 42 (59.2) | 17 (15.9) | 102 (34.0) | 52 (57.8) | 78 (39.0) |
| No | 21 (29.6) | 50 (46.7) | 164 (54.7) | 27 (30.0) | 46 (23.0) |
| Unknown | 8 (11.3) | 40 (37.4) | 34 (11.3) | 11 (12.2) | 76 (38.0) |
| Pre-existing comorbidities – n (%) | | | | | |
| None | 18 (25.4) | 23 (21.5) | 31 (10.3) | 13 (14.4) | 27 (13.5) |
| Hypertension | 51 (71.8) | 70 (65.4) | 258 (86.0) | 76 (84.4) | 160 (80.0) |
| Hyperuricemia | 9 (12.7) | 12 (11.2) | 98 (32.7) | 2 (2.2) | 33 (16.5) |
| Cerebrovascular disease | 0 (0.0) | 4 (3.7) | 26 (8.7) | 0 (0.0) | 3 (1.5) |
| Non-coronary heart disease | 1 (1.4) | 14 (13.1) | 5 (1.7) | 1 (1.1) | 5 (2.5) |
| Coronary diseases | 3 (4.2) | 4 (3.7) | 3 (1.0) | 2 (2.2) | 7 (3.5) |
| No concomitant medication prescribed – n (%) | 16 (22.5) | 8 (7.5) | 25 (8.3) | 2 (2.2) | 16(8.0) |
| Antihypertensive agents – n (%) | 54 (76.1) | 76 (71.0) | 240 (80.0) | 79 (87.8) | 155 (77.5) |
| ARBs | 37 (52.1%) | 65 (60.8%) | 213 (71.0%) | 36 (40.0%) | 26 (13.0%) |
| DPH CCBs | 9 (12.7) | 48 (44.9) | 101 (33.7) | 68 (75.6) | 55 (27.5) |
| Beta blockers | 11 (15.5) | 23 (21.5) | 72 (24.0) | 43 (47.8) | 18 (9.0) |
| ACE inhibitors | 20 (28.2) | 1 (0.9) | 17 (5.7) | 7 (7.8) | 72 (36.0) |
| Diuretics | 15 (21.1) | 5 (4.7) | 4 (1.3) | 2 (2.2) | 26 (13.0) |
| Non DPH CCBs | 4 (5.6) | 0 (0.0) | 2 (0.7) | 10 (11.1) | 155 (77.5) |
| Uric acid lowering agents – n (%) | 5 (7.0) | 23 (21.5) | 72 (24.0) | 43 (47.8) | 18 (9.0) |
| Lipid-lowering agents – n (%) | 15 (21.1) | 32 (29.9) | 123 (41.0) | 2 (2.2) | 43 (21.5) |

*Abbreviations*: BMI, body mass index; BP, blood pressure; DM, diabetes mellitus; ADPKD, autosomal dominant polycystic kidney disease; ESKD, end-stage kidney disease; ARBs, Angiotensin II receptor blockers; ACE, angiotensin converting enzyme; DPH CCBs, Dihydropyridine calcium channel blockers;

Table S2. Kidney disease-related characteristics of patients across sites

| Variables | **Turkey**  **(N=71)** | **Taiwan**  **(N=107)** | **Korea**  **(N=300)** | **China**  **(N=90)** | **Australia**  **(N=200)** |
| --- | --- | --- | --- | --- | --- |
| Serum creatinine (mg/dL) | 1.17 ± 0.43 | 1.08 ± 0.42 | 1.12 ± 0.38 | 1.51 ± 0.49 | 1.16 ± 0.43 |
| eGFR (mL/min/1.73m^2^) | 69.9 ± 30.3 | 70.8 ± 28.0 | 65.8 ± 26.0 | 56.0 ± 25.8 | 75.5 ± 30.5 |
| Blood urea nitrogen (mg/dL) | 18.8 ± 6.8 | 18.8 ± 6.2 | 16.6 ± 5.5 | 20.1 ± 6.9 | 23.1 ± 26.1 |
| Uric acid – mg/dL | 5.6 ± 1.7 | 5.8 ± 2.2 | 5.5 ± 1.3 | 6.1 ± 1.8 | 6.4 ± 1.8 |
| Urine PCR (mg/mg) | 206.2 ± 305.3 | 3.5 ±  4.9 | 146.6 ± 244.0 | 1091.6 ± 693.9 | 84.9 ± 177.9 |
| CKD Stage – n (%) |  |  |  |  |  |
| Stage 1 | 33 (46.5) | 35 (32.7) | 98 (32.7) | 9 (10.0) | 54 (27.0) |
| Stage 2 | 17 (23.9) | 39 (36.5) | 109 (36.3) | 27 (30.0) | 67 (33.5) |
| Stage 3A | 13 (18.3) | 20 (18.7) | 43 (14.3) | 15 (16.7) | 43 (21.5) |
| Stage 3B | 8 (11.3) | 13 (12.2) | 50 (16.7) | 39 (43.3) | 36 (18.0) |
| MIC |  |  |  |  |  |
| Class 1A | 1 (1.4) | 0 (0.0) | 25 (8.3) | 0 (0.0)) | 0 (0.0) |
| Class 1B | 2 (2.8) | 0 (0.0) | 79 (26.3) | 0 (0.0) | 20 (10.0) |
| Class 1C | 5 (7.0 | 0 (0.0) | 93 (31.0) | 24(26.7) | 27 (13.5) |
| Class 1D | 4 (5.6 | 0 (0.0) | 62 (20.7) | 0 (0.0) | 16 (8.0) |
| Class 1E | 3 (4.2) | 0 (0.0) | 26 (8,7) | 0 (0.0) | 7 (3.5) |
| HtTKV(mL/m) | 1073.5 ± 823.5 |  | 1031.4 ± 679.3 | 880.0 ± 687.2 | 925.4 ± 567.4 |
| Kidney length by ultrasound (cm) | 20.0 | 13.1 ± 2.7 |  |  | 16.5 ± 4.5 |
| Genetic test results |  |  |  |  |  |
| *PKD1 PT* mutation | 0 (0.0) | 0 (0.0) | 117 (39.0) | 0 (0.0) | 1 (0.5) |
| *PKD1 NT* mutation | 0 (0.0) | 0 (0.0) | 66 (22.0) | 0 (0.0) | 0 (0.0) |
| *PKD2* mutation | 1 (1.4) | 0 (0.0) | 47 (15.7) | 0 (0.0) | 0 (0.0) |
| Mutation not found | 0 (0.0) | 0 (0.0) | 24 (8.0) | 0 (0.0) | 0 (0.0) |
| Unknown | 70 (98.6) | 107 (100.0) | 46 (15.3) | 90 (100.0) | 199 (99.5) |
| PROPKD score |  |  |  |  |  |
| 0-3 | 0 (0.0) | 0 (0.0) | 64 (21.3) | 0 (0.0) | 0 (0.0) |
| 4-6 | 1 (1.4) | 0 (0.0) | 68 (22.7) | 0 (0.0) | 1 (0.5%) |
| 7-9 | 0 (0.0) | 0 (0.0) | 47 (15.7) | 0 (0.0) | 0 (0.0) |
| Missing | 70 (98.6) | 107 (100.0) | 121 (40.3%) | 90 (100.0) | 199 (99.5) |

*Abbreviations*: eGFR, estimated glomerular filtration rate; CKD, chronic kidney disease; PCR, protein creatinine ratio; htTKV, height-adjusted total kidney volume; MIC, Mayo Imaging Classification; *PKD1 PT*, *PKD1* protein truncating; *PKD1 NT*, *PKD1* nontruncating

Table S3. Criterion for patients deemed to be in rapid progressor

| **Criterion for RP ADPKD** | **All RPs** **(n=426)** |
| --- | --- |
| eGFR decline in one year†, N (%) | 113 (26.5) |
| eGFR decline over five years‡, N (%) | 12 (2.8) |
| Historical htTKV growth§, N (%) | 2 (0.5) |
| MIC 1C-1E, N (%) | 174 (40.9) |
| Kidney length (> 16 cm), N (%) | 4 (0.9) |
| *PKD1 PT* mutation and PROPKD score (>6), N (%) | 1 (0.2) |
| Two criteria, N (%) | 109 (25.6) |
| Three Criteria, N (%) | 11 (2.6) |

*Note*: %, proportion of patients to total number of eligible patients

†, ≥5 mL/min/1.73 m^2^; ‡, ≥2.5 mL/min/1.73 m^2^/yr; §, htTKV ≥5%/yr

*Abbreviations*: ADPKD, autosomal dominant polycystic kidney disease; htTKV, height-adjusted total kidney volume; MIC, Mayo Imaging Classification; *PKD1 PT*, *PKD1* protein truncating; RP, rapid progressor

Table S4. Cox proportional hazards model for outcome event (End-stage kidney disease)

| Variables | **Hazards ratio** | **95% CI** | | p-value |
| --- | --- | --- | --- | --- |
| RP (Ref: SP) | 4.04 | 1.12 | 14.61 | 0.033 |
| Age | 0.95 | 0.92 | 0.99 | 0.006 |
| Systolic blood pressure | 1.03 | 1.00 | 1.05 | 0.028 |

*Abbreviations*: CI, confidence interval; RP, Rapid progressor; SP, Slow progressor

Adjusted for progression groups, age, chronic kidney disease stage, gender, family history of ADPKD reaching end-stage kidney disease, systolic blood pressure diastolic blood pressure, diabetes mellitus, *PKD* mutation
